# RAD51 Inhibitor Reverses Etoposide-Induced Genomic Toxicity and Instability in Esophageal Adenocarcinoma Cells

**DOI:** 10.46439/toxicology.2.006

**Published:** 2020

**Authors:** Chengcheng Liao, Jiangning Zhao, Subodh Kumar, Chandraditya Chakraborty, Srikanth Talluri, Nikhil C. Munshi, Masood A. Shammas

**Affiliations:** 1 Dana Farber Cancer Institute, USA; 2 Veterans Administration Boston Healthcare System, USA; 3 Harvard Medical School, USA

**Keywords:** Genomic instability, Genomic evolution, Homologous recombination, RAD51, Etoposide, Chemotherapy, Esophageal adenocarcinoma

## Abstract

**Aim:**

In normal cells, homologous recombination (HR) is strictly regulated and precise and plays an important role in preserving genomic integrity by accurately repairing DNA damage. RAD51 is the recombinase which mediates homologous base pairing and strand exchange during DNA repair by HR. We have previously reported that HR is spontaneously elevated (or dysregulated) in esophageal adenocarcinoma (EAC) and contributes to ongoing genomic changes and instability. The purpose of this study was to evaluate the impact of RAD51 inhibitor on genomic toxicity caused by etoposide, a chemotherapeutic agent.

**Methods:**

EAC cell lines (FLO-1 and OE19) were cultured in the presence of RAD51 inhibitor and/or etoposide, and impact on cell viability, apoptosis and genomic integrity/stability investigated. Genomic integrity/stability was monitored by evaluating cells for γ-H2AX (a marker for DNA breaks), phosphorylated RPA32 (a marker of DNA end resection which is a distinct step in the initiation of HR) and micronuclei (a marker of genomic instability).

**Results:**

Treatment with etoposide, a chemotherapeutic agent, was associated with marked genomic toxicity (as evident from increase in DNA breaks) and genomic instability in both EAC cell lines. Consistently, the treatment was also associated with apoptotic cell death. A small molecule inhibitor of RAD51 increased cytotoxicity while reducing genomic toxicity and instability caused by etoposide, in both EAC cell lines.

**Conclusion:**

RAD51 inhibitors have potential to increase cytotoxicity while reducing harmful genomic impact of chemotherapy.

## Introduction

Genomic instability, the ability to constantly acquire changes in the sequence and structure of chromosomal DNA, is a common feature of most cancers [1-3]. There is now substantial evidence that the genome of a cell becomes unstable at some early stage during oncogenesis [4-6]. This allows precancerous cells to acquire a variety of new characteristics, some of which then contribute to oncogenic transformation and subsequent progression to advanced disease states and fatal outcome [7,8]. A striking genomic instability has also been observed in esophageal adenocarcinoma (EAC), cancer-associated with gastro esophageal reflux disease. Prolonged exposure to acid and bile in the refluxate can lead to a precancerous lesion known as Barrett’s esophagus (BE), which gradually progresses to EAC, cancer with poor survival rate [9]. There is substantial evidence that genomic instability on EAC arises prior to oncogenesis and enables precancerous cells to constantly acquire genomic changes that underlie the development of cancer and subsequent progression to advanced stages of disease [10]. Consistent with this, the genetic changes are not only detected in EAC cases but also in BE [11]. In fact, all sorts of genomic changes have been detected in BE cases. These include aneuploidy which increases with progression to cancer [12], instability at microsatellite DNA sequences [13], copy number events which increase in number and size of DNA fragment involved [5] and other genetic as well as epigenetic changes [14]. The overall mutational burden in BE could be even higher than in certain cancers [15,16]. Therefore, it is quite evident that genomic instability in EAC occurs at a precancerous state and contributes to its progression to EAC.

Aberrant genomic landscape [15], marked tumor heterogeneity and frequent development of drug resistance [17] in EAC suggest that genomic instability which arises at precancerous state, persists following oncogenic transformation. Furthermore, the evaluation of EAC patient genomes by sequencing suggests that genomic instability increases with progression [15]. There is also substantial evidence that genomic instability underlies progression [18] and associated with poor clinical outcome [19]. It is, therefore, extremely important that we understand the mechanisms underlying genomic instability and develop strategies to inhibit/reduce the acquisition of new genomic changes over time.

Data from our laboratory show that homologous recombination (HR), known to be the most precise DNA repair system, is spontaneously elevated and thus dysregulated in EAC and multiple myeloma. The dysregulated HR is not only involved in genomic instability [20-22] and development of resistance to treatment [22] but also in tumor growth *in vivo* [23]. Therefore, inhibition of HR, whether mediated chemically or by transgenic manipulations, inhibits genomic instability and the ability of EAC cells to grow as tumors. Data from our laboratory also show that exposure of human cells to bile and acid, which are the major components of gastro esophageal refluxate, increases DNA damage and HR activity [20]. Therefore, exposure to acid and bile could also be attributed to elevated HR and genomic instability in EAC. Since most chemotherapeutics are DNA breaking agents, chemotherapy can further increase HR and genomic instability in EAC. In this study, we demonstrate that a chemotherapeutic agent (etoposide) increases DNA damage and genomic instability in EAC cells, and this is reversed by treatment with recombinase (RAD51) inhibitor.

## Materials and Methods

### Cell types

EAC cell lines (FLO-1 and OE19) were purchased from Sigma Aldrich Corporation (Saint Louis, MO) and cultured as described previously [20-24].

### Chemicals

RAD51 Inhibitor [RI-1; 3-chloro-1-(3,4-dichlorophenyl)-4-morpholino-1H-pyrrole-2,5-dione] was purchased from Calbiochem and etoposide [25,26], a chemotherapeutic agent, purchased from Tocris.


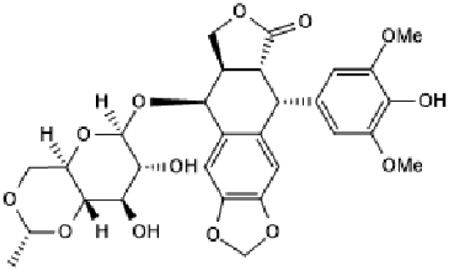


### Etoposide

[5R-[5α, 5aβ,8aα,9β(R*)]]-9-[(4,6-O-Ethylidene-β-D-glucopyranosyl)oxy]-5,8,8a,9-tetrahydro-5-(4-hydroxy-3,5-dimethoxyphenyl)furo[3’,4’:6,7]naphtho[2,3-d]-1,3-dioxol-6-(5aH)-one [25,26].

### Cell viability

Cell Titer-Glo Luminescent Viability Assay kit (Promega Corporation, Madison, WI) was used to assess cell viability.

### Apoptosis assay

FLO1 and OE19 cells, control or those treated with RAD51 inhibitor and/or etoposide, were washed and treated with FITC (fluorescein isothiocyanate)-conjugated Annexin V and propidium iodide. The percentage of cells undergoing apoptosis in each treatment cohort was analyzed by flow cytometry.

### DNA breaks and DNA end resection

Impact of treatments on DNA breaks was assessed by evaluating cells for DNA break marker (γ-H2AX) and DNA end resection (a distinct step in the initiation of HR) monitored by investigating phosphorylation of RPA32 on ser4 and ser8 [27].

### Homologous Recombination Assay

Homologous recombination (HR) activity was assessed using a luminescence-based functional assay as reported by us previously [20,23].

### Genomic instability

Genomic instability was monitored by evaluating cells for micronuclei, a marker of genomic instability [28], using a commercial kit as described by us [29].

## Results

### RAD51 inhibitor inhibits homologous recombination activity in EAC cells

**Figure 1:**
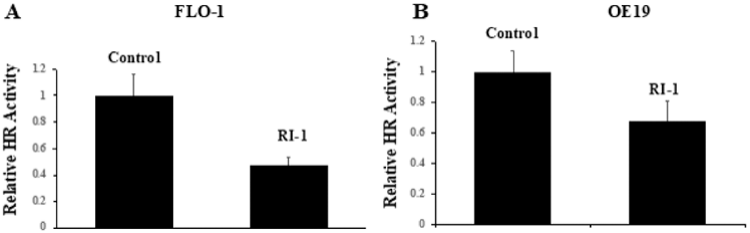
RAD51 inhibitor inhibits homologous recombination in EAC cells. EAC cell lines (FLO1 and OE19), control or those treated with RAD51 inhibitor (RI-1; 20 μM) for 48 hrs., were evaluated for homologous recombination (HR) activity, using a plasmid-based functional assay as described in Methods. Bar graphs showing HR activity in FLO-1 (A) and OE19 (B) cells; Error bars represent SDs of three independent experiments.

We have shown that the inhibition of recombinase (RAD51), whether mediated by transgenic suppression [20,22] or chemical manipulation [23], inhibits homologous recombination (HR) activity. Here, we used the RAD51 inhibitor “RI-1” and show that it inhibits HR activity in both EAC cell lines tested (Figure 1).

### RAD51 inhibitor reverses etoposide-induced DNA damage in EAC cells

**Figure 2:**
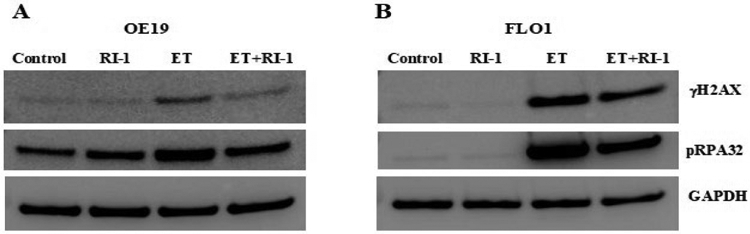
RAD51 inhibitor reverses etoposide-induced DNA damage in EAC cells. EAC cell lines (OE19 and FLO1), control (C) or those treated with RAD51 inhibitor (RI-1; 20 μM), etoposide (ET; 1 μM) and combination of RAD51 inhibitor and etoposide for 48 hrs., were evaluated for impact on γH2AX (a marker of DNA breaks) and pRPA32 (a marker of DNA end resection) by Western blotting. Western blot images (A) and bar graph showing protein levels normalized to GAPDH (B) are presented.

EAC cell lines (OE19 and FLO1) were treated with etoposide and RAD51 inhibitor (RI-1), alone as well as in combination with each other for 48 hrs., and evaluated for impact on γH2AX (a marker of DNA breaks) and pRPA32 (a marker of DNA end resection) by Western blotting. Treatment with etoposide increased DNA breaks by ~6-fold and DNA end resection by 2-fold in OE19 cells (Figure 2A). Similarly, in FLO-1 cells, etoposide led to an increase in DNA breaks by ~40-fold and DNA end resection by ~50-fold (Figure 2B). These data show that etoposide causes massive DNA damage in EAC cells. When etoposide was combined with recombinase (RAD51) inhibitor “RI-1”, the etoposide-induced DNA breaks were inhibited by 50% and DNA end resection reduced by ~30% in OE19 cells. RAD51 inhibitor also caused a reduction in etoposide-induced DNA breaks and DNA end resection in another cell line (FLO-1; Figure 2B). These data show that chemotherapeutic agent etoposide causes a massive DNA damage which can be reduced by RAD51 inhibitor.

### RAD51 inhibitor inhibits etoposide-induced genomic instability in EAC cells:

**Figure 3:**
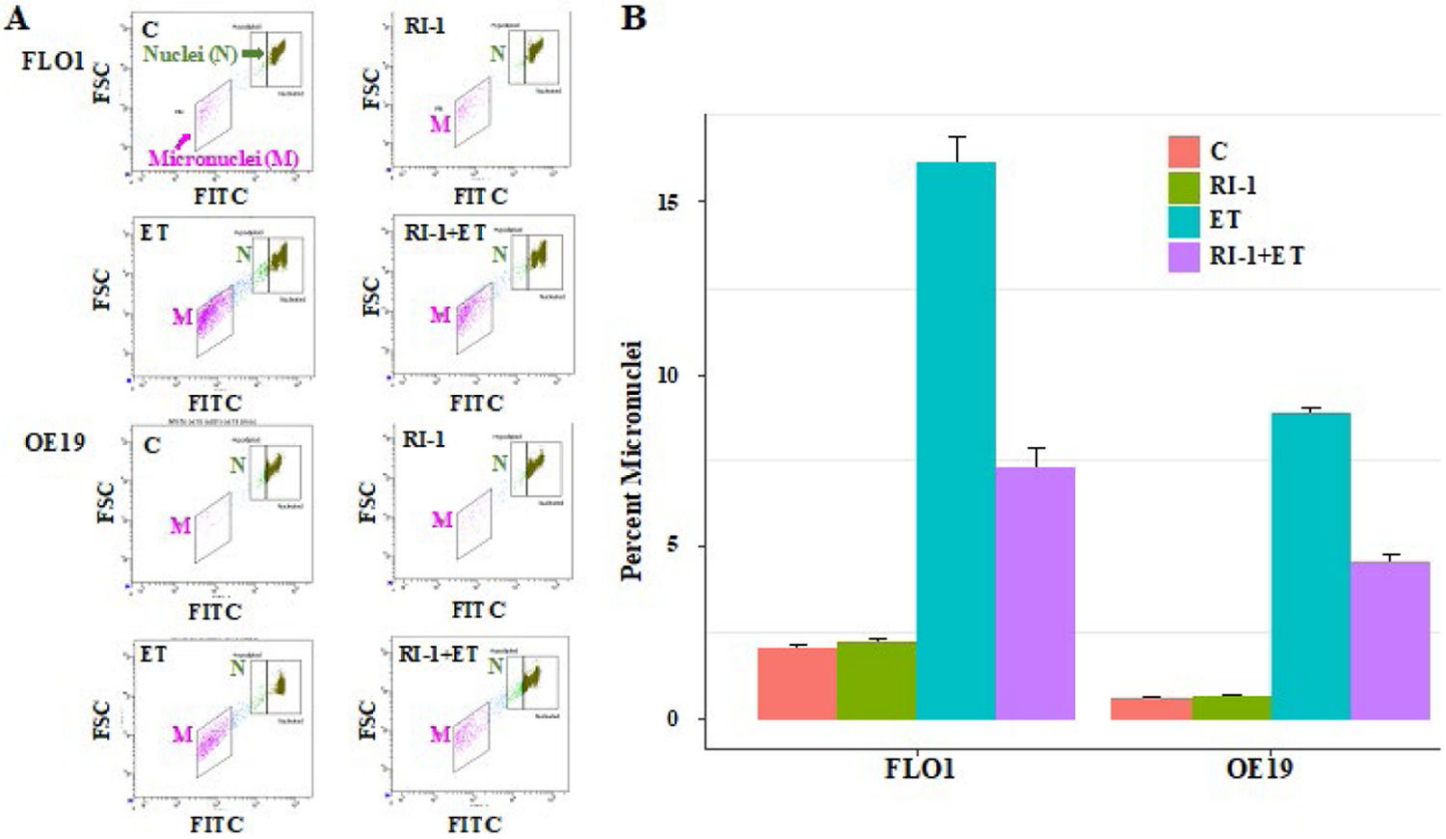
RAD51 inhibitor inhibits etoposide-induced genomic instability in EAC cells. EAC (FLO-1 and OE19) cell lines, control (C) or those treated with RAD51 inhibitor (RI-1; 20 μM), etoposide (ET; 1 μM) and combination of RAD51 inhibitor and etoposide for 48 hrs., were evaluated for impact on micronuclei (a marker of genomic instability). Flow cytometry images of micronuclei **(A)** and bar graphs showing percentage of micronuclei **(B)** are shown.

We also evaluated the impact of etoposide and/or RAD51 inhibitor “RI-1” on genomic instability in EAC (FLO-1 and OE19) cell lines. Control and treated cells were cultured for 48 hrs., and impact on genome stability monitored by evaluating micronuclei, a marker of genomic instability. Treatment with etoposide was responsible for 8-fold and 15-fold (p<0.003) increase in the percentage of micronuclei in FLO-1 and OE19 cells, respectively (Figure 3). When etoposide was combined with RAD51 inhibitor, the etoposide-induced micronuclei were reduced by ~50% (p<0.02) in both cell lines (Figure 3). These data show that etoposide increases genomic instability in EAC cells which is reversed/reduced by RAD51 inhibitor.

Flow cytometry images of micronuclei (A) and bar graphs showing percentage of micronuclei (B) are shown.

### RAD51 inhibitor synergistically increases the cytotoxicity of etoposide in EAC cells

Next evaluated the impact of RAD51 inhibitor on etoposide-induced cytotoxicity in EAC cells. EAC (FLO-1) cells were treated with different concentrations of RAD51 inhibitor “RI-1” and/or etoposide for 48 hrs., and cell viability assessed as described in Methods. RAD51 inhibitor increased the cytotoxicity of etoposide (Figure 5A). Combination index plots (Figure 5B) show that an increase in etoposide-induced cytotoxicity by RAD51 inhibitor is synergistic. These data show that RAD51 inhibitor inhibits EAC cell growth and synergistically increases that the efficacy of chemotherapeutic agent etoposide.

### RAD51 inhibitor increases apoptotic cell death by etoposide in EAC cells

**Figure 4:**
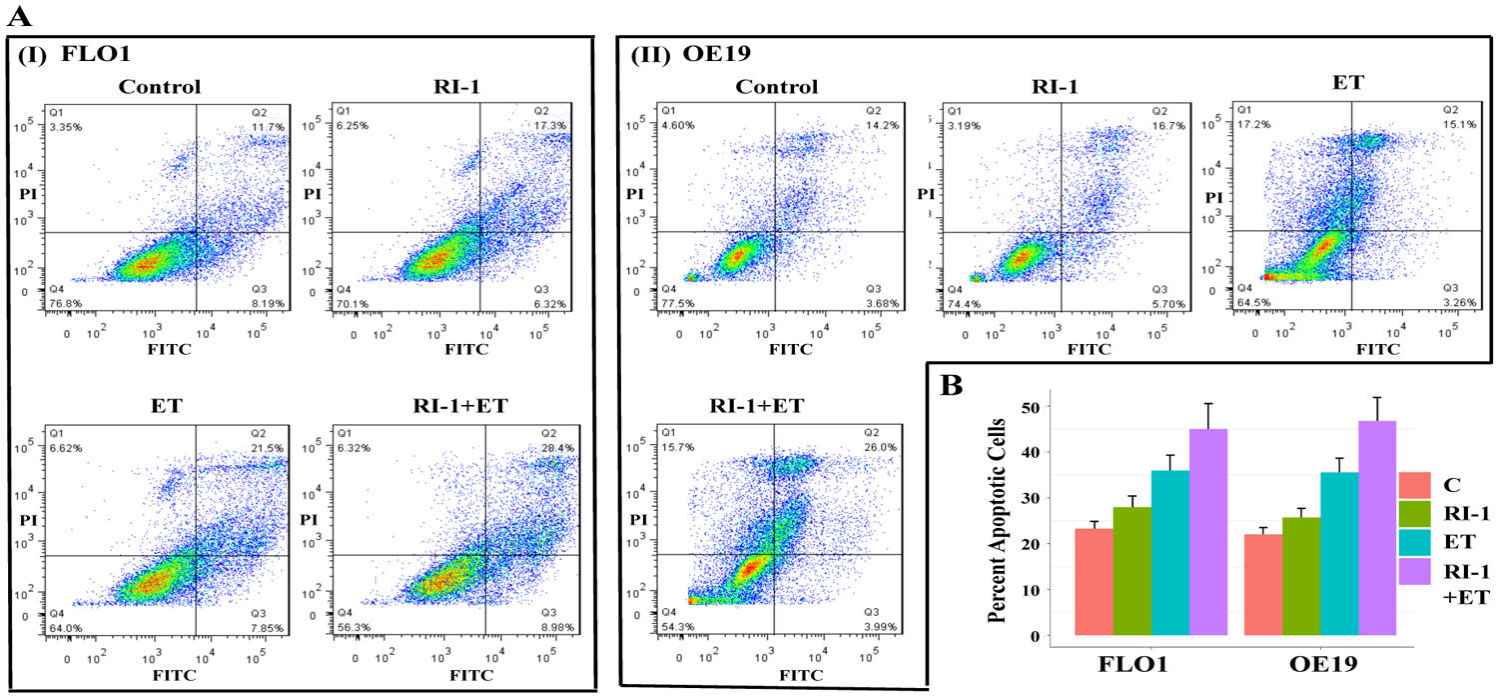
RAD51 inhibitor increases etoposide-induced apoptosis in EAC cells. EAC (FLO-1 and OE19) cell lines, control (C) or those treated with RAD51 inhibitor (RI-1; 20 μM), etoposide (ET; 1 μM) and combination of RAD51 inhibitor and etoposide for 48 hrs., were evaluated for apoptosis using flow cytometry. (A) Flow cytometry images of FLO-1 (I) and OE19 (II) cells; (B) Bar graphs showing percentage of apoptotic cells; Errors bars represent SDs of triplicated assays.

**Figure 5:**
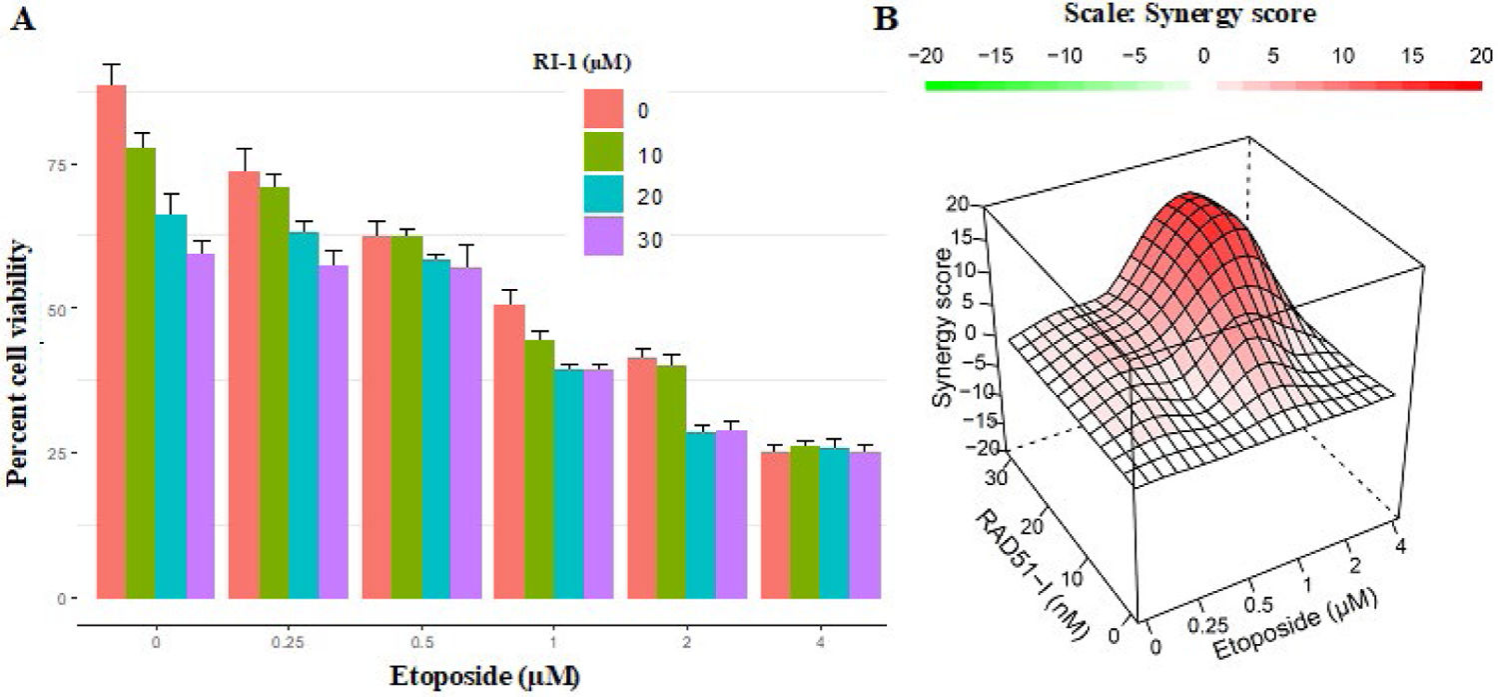
RAD51 inhibitor increases cytotoxicity of etoposide in EAC cells. EAC (FLO-1) cells were treated with different concentrations of RAD51 inhibitor (RI-1) and etoposide for 48 hrs., and cell viability assessed as described in Methods. (A) Bar graph showing percent cell viability; Error bars represent SDs of triplicate assays; (B) Combination index visualized in R environment using the HSA method in the synergy-finder package. A score more than 0 (red) indicates a synergistic effect of the combination.

To investigate the mechanism of cell death, EAC cell lines were treated with etoposide and RAD51 inhibitor “RI-1”, alone as well as in combination with each other, for 48 hrs., and evaluated for apoptosis by annexin labeling. Treatment with both the RAD51 inhibitor and etoposide caused apoptotic cell death in EAC cells. Relative to control FLO-1 cells, the treatment with RAD51 inhibitor, etoposide and combination increased the percentage of apoptotic cells by 4.7 (± 1.56), 12.8 (± 2.39) and 21.8 (± 4.89), respectively. Similarly, in OE19 cells the treatment with RAD51 inhibitor, etoposide and combination increased the percentage of apoptotic cells by 3.6 (± 1.30), 13.5 (± 1.69), and 24.7 (± 4.37), respectively (Figure 4). Thus, both the RAD51 inhibitor and etoposide caused apoptotic cell death in EAC cell lines and RAD51 inhibitor increased the percentage of cells undergoing etoposide-induced apoptosis in both EAC cell lines.

## Discussion

EAC is characterized by a marked genomic instability [10,11,15,16]. Consistently, the cancer patients display striking tumor heterogeneity [15] and frequently develop chemo resistance [17]. There is substantial evidence that genomic instability in EAC appears at a precancerous state and intensifies with progression to advanced disease states. Genomic instability, the ability to constantly acquire genomic changes, is considered to play a key role in clonal evolution and disease progression including the development of drug resistance. Consistent with this, the exome sequencing of multiple myeloma patient samples has revealed that an increased number of mutations associates with poor overall and event-free survival of patients [2]. Genomic instability and a resulting increase in the mutational load can also lead to increased levels of neoantigens and other new characteristics that can enable aberrant/transformed and/or cancer cells escape recognition and elimination by immune system [30]. Investigating mechanisms of genomic instability in esophageal adenocarcinoma and multiple myeloma model systems, we have demonstrated that homologous recombination (HR), the only known error-free DNA repair system, is spontaneously elevated/dysfunctional and involved in genomic instability [20-22] and emergence of drug resistance [22].

Chemotherapeutic agents such as etoposide are genotoxic and kill cancer cells by inducing DNA damage/breaks. However, there are several problems associated with chemotherapy. One of the problems is that although most or a large number of cancer cells are killed by chemotherapy, a subset of cells that survive the treatment end up having increased DNA damage/breaks caused by chemotherapy [29]. Since DNA breaks induce HR [31,32], the treatment of cancer cells with a DNA damaging agent can further increase HR activity [22], leading to an increase in genomic instability [22,29]. This is exactly what we observed in this study. We treated EAC cells with etoposide and evaluated adherent (live) cells for markers of DNA breaks and genomic instability. Our data show that etoposide increases DNA breaks and DNA end resection (a distinct step in the initiation of HR) (Figure 2) as well as increases genomic instability (Figure 3) in EAC cells. These data confirm that the treatment with chemotherapeutic agents such as etoposide increases genomic toxicity (as evident from increased DNA breaks) as well as instability (as evident from increased micronuclei) in EAC cells. Etoposide has been shown to induce genomic aberrations in human cells [33]. Consistent with these data, we have also demonstrated that melphalan, which is also a chemotherapeutic agent, further increases homologous recombination activity and genomic instability in human multiple myeloma cells [29].

The increase in genomic toxicity and instability by chemotherapeutic agents, as demonstrated here by etoposide, could also pose a risk of transformation in normal cells of a patient. In the case of cancer cells of a patient, the increased genomic instability/evolution can potentially increase the likelihood and/or reduce time to progression, including the development of drug resistance. This is probably the reason that secondary cancers are observed in some patients treated with certain chemotherapeutic drugs [34,35]. Some reports also suggest that chemotherapy is probably worse than radiation in its contribution to the development of leukemia. Therefore, there is an urgent need to identify/develop drugs that inhibit mechanisms driving genomic evolution in cancer cells. Such treatments will potentially inhibit/reduce spontaneous as well as chemotherapy-induced genomic instability in cancer cells. Based on our previous data showing that elevated HR drives genomic instability and inhibition of recombinase (RAD51) reduces HR and genomic instability [20,22,23], we evaluated the impact of RAD51 inhibitor on etoposide-induced genomic toxicity and instability. We show that the RAD51 inhibitor reduces etoposide-induced DNA breaks as well as genomic instability in both EAC cell lines. Importantly, the RAD51 inhibitor synergistically increased etoposide-induced cell death in EAC cells. These data are consistent with our previous study demonstrating that a small molecule inhibitor of APEX activity, involved in dysregulation of HR and genome stability, increases chemotherapy-induced cell death while reversing/reducing its harmful genomic impact [29]. The question is that how RAD51 inhibitor increases cytotoxicity while reducing genomic instability caused by etoposide. The reason is that EAC cells have increased DNA damage and HR activity. When these cells are treated with etoposide, the cells with extensive DNA damage are killed. A subset of cells that have moderate and/or low DNA damage following etoposide treatment, HR helps them survive by removing DNA breaks. However, the HR, which was already elevated in EAC, is further increased by etoposide and thus leads to an increase in genomic instability in surviving cells. When etoposide is combined with RAD51 inhibitor, HR activity is reduced. This leads to an increase in etoposide-induced cell death (because of loss of DNA break repair by HR) and reduction in genomic instability (which was caused by elevated/unnecessary and imprecise HR). Consistent with this study, our published [29] and unpublished data demonstrate that when HR inhibitors are combined with chemotherapy, the chemotherapy-induced cytotoxicity is increased whereas genomic toxicity and instability are reduced. In summary, we show that small molecule inhibitors of RAD51 have the potential to increase cancer cell killing while reducing/minimizing genomic instability caused by chemotherapy.
